# Distinct dendritic cell cytoskeletal programs dictate synapse architecture and CD8^+^ T cell fate

**DOI:** 10.3389/fimmu.2026.1716644

**Published:** 2026-04-01

**Authors:** Camille D. Clamagirand, Christoph Ratswohl, Marc W. Schmid, Theresia Eich, Nadine Anslinger, Daniel F. Legler, Jérémie Rossy

**Affiliations:** 1 Institute of Cell Biology and Immunology Thurgau (BITG), University of Konstanz, Kreuzlingen, Switzerland; 2 Graduate School for Cellular and Biomedical Sciences, University of Bern, Bern, Switzerland; 3 Department of Biology, University of Konstanz, Konstanz, Germany; 4 MWSchmid GmbH, Glarus, Switzerland; 5 Theodor Kocher Institute, University of Bern, Bern, Switzerland

**Keywords:** actin cytoskeleton, CD70, dendritic cell, immunological synapse, T cell, T cell-DC interactions

## Abstract

Dendritic cell activation of CD8^+^ T cells at the immunological synapse is critical for immunity, but the structural organization of the dendritic cell side and its impact on T cell fate remain poorly defined. Using bone marrow-derived dendritic cells (BMDCs) as a model, we describe two stable subpopulations distinguished by their capacity to form morphologically distinct synapses. We demonstrate that this architectural divergence is governed by the differential expression of the co-stimulatory molecule CD70: CD70^high^ BMDCs form spiky “firework” synapses driven by a filopodia-based cytoskeletal program, while CD70^low^ BMDCs form smooth “pancake” synapses. This structural dichotomy functionally dictates T cell programming. CD70^high^ dendritic cells prime potent, terminally differentiated Tc1 effector cells. In contrast, IL-6-secreting CD70^low^ dendritic cells generate memory T cells with a Tc17-like functional profile and robust recall capacity. Our work reveals that DC synapse architecture is a key determinant of T cell fate, linking the physical organization of the cell to distinct immunological outcomes.

## Introduction

The initiation of adaptive immunity against pathogens and tumors relies on the ability of dendritic cells to process and present antigens to prime naive T cells. As sentinels of the immune system, dendritic cells reside in peripheral tissues where they are activated by pathogen-associated molecular patterns (PAMPs) or inflammatory signals (1). This maturation process is a profound transformation, equipping dendritic cells with the migratory capacity to reach secondary lymphoid organs and upregulating the surface molecules required for effective T cell activation (2, 3). Central to this dialogue between dendritic cell and T cell is the formation of a specialized cell-cell junction known as the immunological synapse, a highly organized and dynamic platform for intercellular communication (4).

While dendritic cell maturation alone provides a baseline capacity for T cell priming, the generation of a robust cytotoxic CD8^+^ T cell response often requires an additional layer of activation known as “licensing” (5, 6). This process is typically mediated by cognate interactions with CD4^+^ T helper cells, where the engagement of CD40 on the dendritic cells by CD40L on the T cell provides a critical signal (7). A licensed dendritic cell is functionally upgraded, expressing higher levels of costimulatory ligands that are essential to overcome the higher activation threshold of naive CD8^+^ T cells and to drive their clonal expansion and differentiation into cytotoxic effectors (8). This enhancement is largely mediated by members of the Tumor Necrosis Factor Superfamily (TNFSF) (9).

Among these, the costimulatory molecule CD70 has emerged as a particularly critical player in CD8^+^ T cell immunity. Upregulated on dendritic cells following CD40-mediated licensing (10), CD70 engages its receptor, CD27, on T cells to provide potent signals that promote T cell survival, proliferation, and, crucially, the development of long-term memory populations (11–13). Indeed, the ability of DCs to prime robust CD8+ T cell responses, particularly in the absence of classical T cell help, has been shown to be critically dependent on the level of CD70 induced by either CD40, TLR stimulation, or alternative CD40-independent licensing pathways (14, 15). The level of CD70 expression on the DC surface can therefore be seen as a key determinant of the quality and durability of the resulting CD8^+^ T cell response, yet how heterogeneity in CD70 expression is regulated and functionally interpreted at the synapse is not fully understood.

The immunological synapse is far more than a simple site of ligand-receptor binding; it is a highly organized structural assembly. On the T cell side, signaling is known to be driven by the nanoscale clustering and microscale spatial patterning of receptors, which are actively organized by the cytoskeleton (16, 17). Synapse architecture itself is not invariant; the classic “bull’s-eye” pattern observed at the B cell-T cell synapse contrasts with the more fragmented, multifocal organization typically seen at the dendritic cell-T cell interface (18). This diversity in form strongly implies a diversity in function, a principle well-established in other fields such as neurobiology, where the precise morphology of a synapse dictates the strength and nature of information transfer (19, 20). However, how the physical architecture of the dendritic cell synapse contributes to the information exchanged with the T cell remains largely unexplored.

Dendritic cells themselves are not a uniform population but comprise functionally specialized subsets (e.g., cDC1, cDC2) and exist within a continuum of differentiation states that are constantly reshaped by their environment (21, 22). It is now understood that the specific function of these subsets is intrinsically linked to their location within secondary lymphoid organs; in essence, location dictates function (21). Different dendritic cell subsets possess distinct migratory abilities that guide them to specific niches—such as the deep T cell zone for cDC1s or the T cell-B cell border for cDC2s —and these locations are optimized for priming different T cell lineages. These migratory patterns and the resulting synapse locations are, in turn, governed by the underlying organization of the dendritic cell cytoskeleton (23). It is therefore plausible that this functional heterogeneity extends to their synapse-building capacity, allowing different dendritic cell subsets to form physically distinct synapses. Such structural differences could represent a novel layer of regulation in T cell priming.

Ultimately, the dendritic cells orchestrate T cell fate, driving differentiation into effector or memory lineages through a combination of co-stimulatory signals and secreted cytokines (24). The intricate relationship between dendritic cell location, synapse structure, and T cell function suggests that the physical architecture of the synapse itself—dictated by the specific dendritic cell subset involved—could be a key factor in this decision-making process. Here, we investigate this hypothesis by exploring the link between synapse architecture in a bone marrow-derived dendritic cell (BMDC) model, the underlying cytoskeletal programs, and the resulting T cell response. We show that these BMDCs comprise two stable subpopulations, first distinguished by their capacity to form morphologically and functionally distinct synapses. We demonstrate that this architectural divergence is driven by distinct, hardwired cytoskeletal programs that correlate with differential CD70 expression, and which in turn program divergent CD8^+^ T cell effector and memory fates.

## Results

### BMDCs form two synapse phenotypes governed by a CD70 signaling threshold

Dendritic cells are the main antigen-presenting cells activating T cells in secondary lymphoid organs during immune responses. They do so through the formation of the immunological synapse, a complex cell-cell interface (4). The T cell side of the immunological synapse has been extensively investigated both for CD4^+^ and CD8^+^ T cells. However, we have a restricted understanding of the dendritic cell side of the synapse, and we know even less of the impact of the T cell type – CD4^+^ or CD8^+^ – on how it is organized and functions.

**Figure 1:**
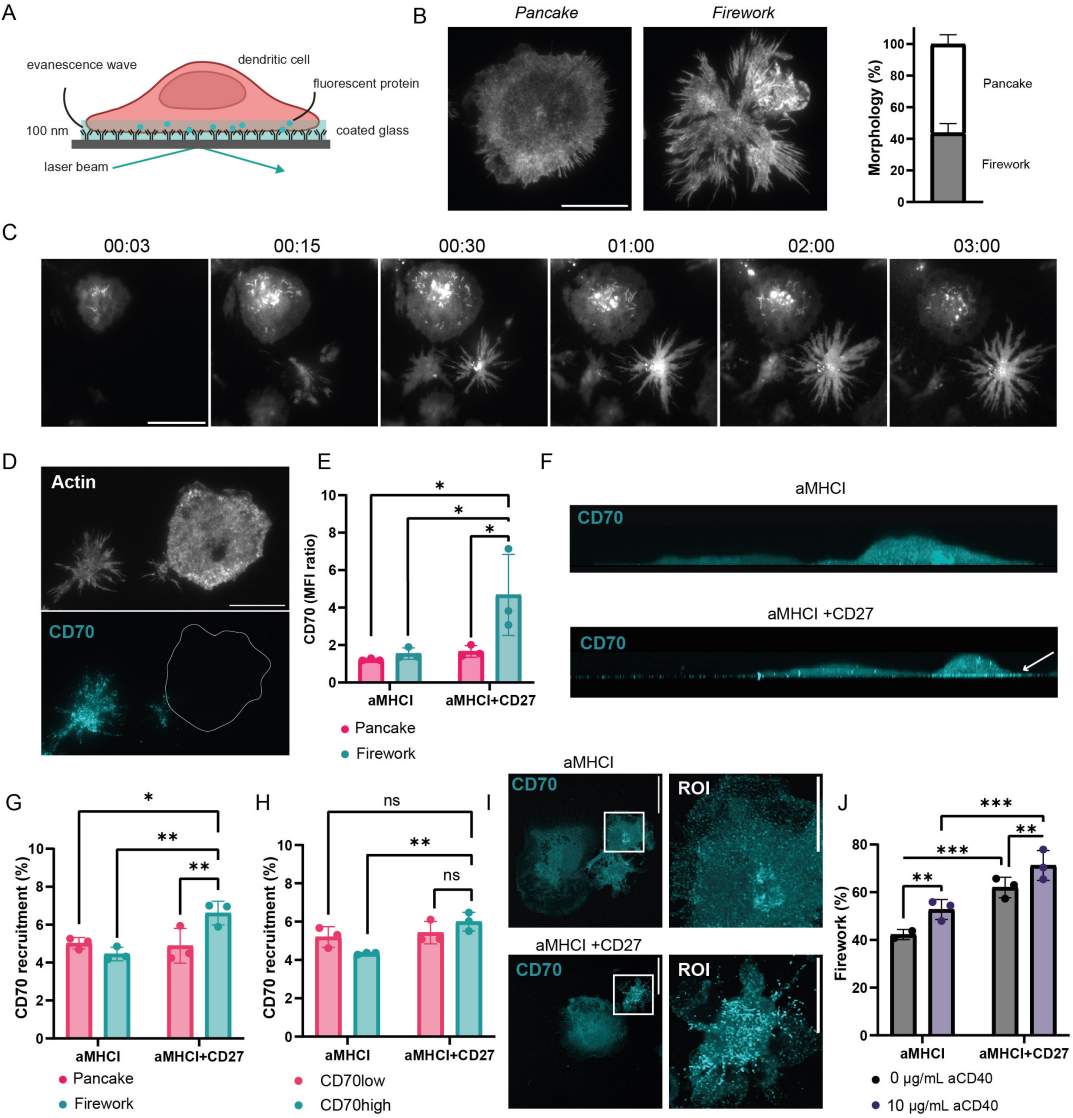
Dendritic cells form distinct synapse morphologies dictated by CD70 signaling. **(A)** Schematic of the pseudo-synapse model using total internal reflection fluorescence (TIRF) microscopy. **(B)** Representative TIRF images BMDCs forming either pancake or firework synapses on anti-MHCI-coated glass, stained for F-actin and quantification of the prevalence of each phenotype (n=4 biological replicates, n_1_ = 127 cells, n_2_ = 155 cells, n_3_ = 24 cells, n_4_ = 168 cells). **(C)** Time-lapse TIRF imaging of a live BMDC stained with CellTracker Deep Red, showing the formation and stability of either pancake or firework synapse. Image acquisition was initiated immediately after the stained BMDCs were added to the imaging chambers. Images were acquired every minute for 3 hours at 37 °C in a 5% CO_2_ atmosphere. Time stamp is hh:mm. **(D)** Representative TIRF images showing CD70 and actin expression in pancake vs. firework synapses on MHCI-coated glass. **(E)** Quantification of membrane-proximal CD70 mean fluorescence intensity (MFI) at the synapse interface (measured by TIRF microscopy) in each synapse phenotype on glass coated with anti-MHCI (aMHCI) alone or with CD27. MFI is normalized to the background (n_1_ = 117 cells, n_2_ = 109 cells, n_3_ = 104 cells). **(F)** Representative confocal images with orthogonal side-views **(G)** showing CD70 localization in BMDCs. The arrow indicates CD70 recruitment to the synapse upon CD27 engagement. **(G)** Quantification of CD70 recruitment to the synapse measured measure by confocal microscopy. Briefly, CD70 mean fluorescence intensity (MFI) was quantified at the synapse and normalized by the CD70 MFI in the whole cell. Cells were classified by their synapse morphologies, namely fireworks or pancakes (n_1_ = 41 cells, n_2_ = 44 cells, n_3_ = 39 cells). **(H)** Quantification of CD70 recruitment to the synapse measured measure by confocal microscopy. Briefly, CD70 mean fluorescence intensity (MFI) was quantified at the synapse and normalized by the CD70 MFI in the whole cell. Cells were classified by their total CD70 expression. To define CD70^high^ and CD70^low^, the median fluorescence intensity was calculated across all cells pooled from three independent experiments, with cells above the median classified as CD70^high^ and cells below as CD70^low^ (n_1_ = 41 cells, n_2_ = 44 cells, n_3_ = 39 cells). **(I)** Representative confocal images showing CD70 distribution in BMDCs. Images are representative of 3 independent experiments. **(J)** Frequency of firework synapses in BMDCs, with or without pre-treatment with anti-CD40 (aCD40), on surfaces coated with aMHCI alone or with CD27 (n_1_ = 547 cells, n_2_ = 629 cells, n_3_ = 1028 cells). Data are shown as mean ± SD (n=3 biological replicates). *p ≤ 0.05; **p ≤ 0.01; ***p ≤ 0.00; ns, non-significant. Main scale bars: 20 µm, inlets scale bars = 10µm. Statistical significance was determined using two-way ANOVA. Note: These images illustrate the two distinct morphological states initially observed in LPS-matured BMDCs. The dendritic cell identity and subpopulation characteristics corresponding to these morphologies are validated by marker analysis (including Zbtb46) in **Figures 2D, E**.

To investigate the structural and molecular organization of the dendritic cell side of the immunological synapse with CD8^+^ T cells, we used a simplified pseudo-synapse model (plating cells on a planar substrate coated with an activating antibody) involving LPS-matured bone marrow-derived dendritic cells (BMDCs) plated onto glass cover slips coated with a functional antibody against MHCI (**Figure 1A**). These LPS-matured cultures were confirmed to be bona fide dendritic cells (CD11c+/MHCII+) and distinct from macrophage populations, as will be demonstrated by definitive transcription factor analysis in the subsequent section. Upon fixation 30 min after having introduced the BMDCs on the cover glass and phalloidin staining for F-actin we observed, in TIRF (total internal reflection fluorescence) microscopy, the formation of two clearly distinct synapse morphologies on the glass: 56.09% ± 5.93% of the cells displayed a round, well-spread morphology with smooth edges, which we arbitrarily named “pancakes,” while the other: 43.80% ± 5.81% of cells exhibited an irregular, star-like shape with filamentous, spiky projections that we defined as “fireworks” (**Figure 1B**). Because we observed these distinct morphologies in fixed cells, we questioned whether they represented different cell populations or whether BMDCs transitioned dynamically between these forms, potentially reflecting different phases of cell spreading. Live-cell imaging of BMDCs stained with the CellTracker Deep Red dye and interacting with anti-MHCI showed that both pancake and firework cells maintained their characteristic synaptic structures for several hours once they were formed (**Figure 1C**, **Supplementary Movie 1**), suggesting the decision dictating synapse morphology is a stable, early event in synapse formation rather than a transient state.

Co-stimulatory molecules, notably members of the TNF superfamily, are essential to priming of T cells by dendritic cells (9, 25). Among TNFSF ligands, CD70 provides a signal that is critical for CD8^+^ T cell activation, effector functions, and differentiation into memory cells (11, 13, 26, 27).

We therefore stained fixed BMDCs plated on anti-MHCI-coated glass with an antibody against CD70 and imaged them in TIRF microscopy to investigate the link between CD70 and the synapse phenotypes we observed. Strikingly, while firework synapses form on anti-MHCI alone, we observed a strong recruitment of CD70 into these structures only when the glass was also coated with its cognate receptor, recombinant CD27 (**Figures 1D, E**). To understand if the increased signal detected in TIRF reflected a difference in recruitment of CD70 to the synapse or a higher level of expression by the firework subtype, we performed optical sectioning of these cells using a super-resolution NSPARC detector. Z-projections of 3D reconstructions revealed intracellular compartments containing CD70 only in BMDCs forming firework synapses, while signal intensity was universally low in the pancake cells (**Supplementary Figure 1A**). Optical sectioning further confirmed that CD70 was recruited to the plasma membrane at the synapse of the firework cells interacting with glass coated with anti-MHCI and CD27 (**Figures 1F, G**). In contrast, CD40 did not show recruitment in the presence of CD27 (**Supplementary Figures 1B, C**), supporting the specificity of the CD70 redistribution. To further investigate the relationship between global CD70 expression and synaptic recruitment, we divided BMDCs into CD70^high^ and CD70^low^ groups based on their total cellular CD70 fluorescence in confocal, and quantified their relative volumetric polarization to the glass interface. Notably, while both populations exhibited a baseline distribution of CD70, only the CD70^high^ cell showed the capacity to significantly mobilize CD70 to the synapse upon CD27 engagement (**Figure 1H**). In contrast, CD70^low^ cell failed to mount a significant recruitment response to the ligand. This indicates that a high global expression of CD70 is a functional prerequisite for the active, ligand-driven synaptic accumulation required to arm the firework synapse.

Interestingly, CD70 recruitment in firework synapses appears to translate into larger and brighter clusters of CD70 (**Figure 1I**). Of note, CD70 clusters could also be observed in the TIRF images (**Figure 1D**). We observed that adding CD27 to the glass surface altered the commitment of the BMDC population at the point of initial synapse formation. This extrinsic signal significantly increased the percentage of cells that formed firework synapses, thereby shifting the balance of the two morphologies observed across the population (**Figure 1E**).

This finding indicates that the synapse phenotype is not determined solely by a cell’s basal CD70 expression. Instead, it suggests that the total strength of the CD70 signal, a combination of a cell’s intrinsic expression level and extrinsic ligand engagement, dictates its morphological commitment upon contact with the surface. Providing CD27 on the glass appears to deliver a sufficiently strong signal to cause a subpopulation of BMDCs, which would otherwise have committed to from a pancake instead of a firework synapse. This led us to hypothesize that a signaling threshold must be reached to trigger this outcome.

CD70 expression is known to be increased during dendritic cell maturation and licensing, particularly following the triggering of CD40 (6, 10, 28, 29). To directly test this signaling threshold hypothesis, we treated BMDCs with a functional anti-CD40 antibody to boost basal CD70 expression. As predicted, this global increase in intrinsic CD70 levels significantly increased the proportion of cells forming firework synapses, an effect that was further amplified when the extrinsic CD27 signal was also present (**Figure 1J**).

Taken together, these data indicate that the choice of synapse morphology is instructed by the total strength of CD70 signaling. This threshold, achievable through either high intrinsic expression or potent extrinsic ligand engagement, appears to dictate a fundamental choice in cellular mechanics, likely by governing the activation of distinct cytoskeletal programs at the onset of synapse formation.

### CD70 expression level demarcates two functionally distinct subpopulations of mature BMDCs

**Figure 2:**
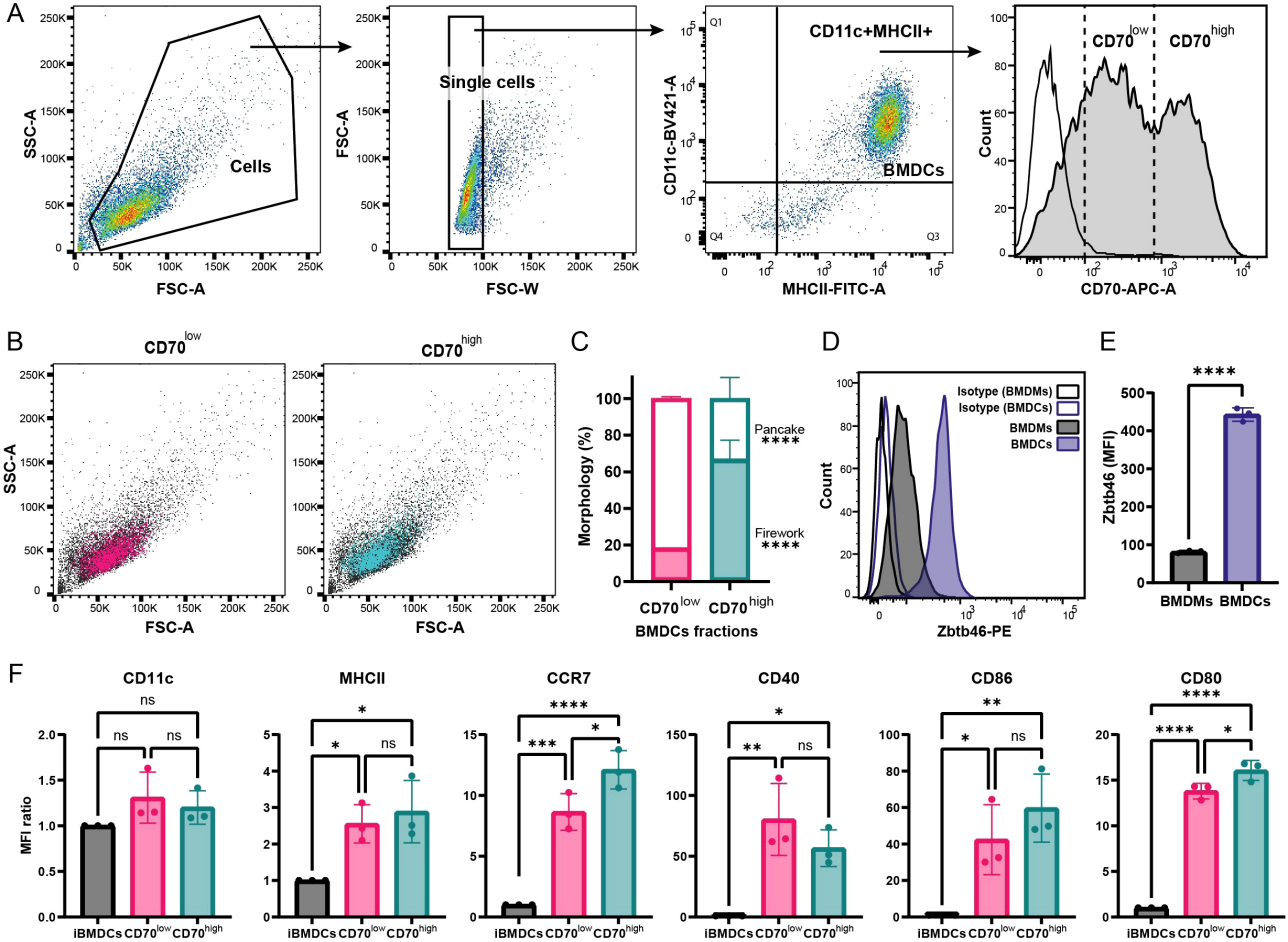
CD70 expression defines two functionally distinct BMDC subpopulations. **(A)** Flow cytometry gating strategy to identify CD70^low^ and CD70^high^ cells within mature BMDCs. **(B)** Forward (FSC) and side scatter (SSC) profiles of the CD70low (magenta) and CD70high (green) populations, showing similar size and granularity. **(C)** Quantification of synapse phenotypes formed by sorted CD70^low^ and CD70^high^ BMDCs on anti-MHCI-coated glass (two-way ANOVA, n_1_ = 328 cells, n_2_ = 323 cells, n_3_ = 257 cells). **(D, E)** Validation of dendritic cell identity. Representative histogram **(D)** and MFI quantification **(E)** of the dendritic cell-specific transcription factor Zbtb46 in BMDCs compared to bone marrow-derived macrophages (BMDMs), (Unpaired Student’s t-test). **(F)** Surface expression of key maturation markers on CD70^low^ vs. CD70^high^ BMDCs, shown as MFI normalized to immature BMDCs (iBMDCs), (two-way ANOVA). Data are shown as mean ± SD (n=3 biological replicates). *p ≤ 0.05; **p ≤ 0.01; ***p ≤ 0.001; ****p ≤ 0.0001; ns, not significant.

Our model predicts that the pancake and firework phenotypes arise from stable BMDC subpopulations distinguished by their basal CD70 expression. We therefore sought to isolate these putative groups to confirm their morphology upon synapse formation. Flow cytometry confirmed the existence of two distinct cell populations in our BMDC cultures based on their level of CD70 expression: CD70^low^ and CD70^high^ cells (**Figure 2A**). Interestingly, both CD70^low^ and CD70^high^ cells appeared morphologically similar based on their side scatter/forward scatter (SSC/FSC) profiles (**Figure 2B**). We then used fluorescence-activated cell sorting (FACS) to separate the two populations. Of note, both populations were for the most majority MHCII^+^ CD11c^+^ (89.07% ± 6.01% for CD70^low^ cells and 91.37% ± 4.45 for CD70^high^ cells, mean ± SD), and the small fraction of MHCII^-^ and/or CD11c^-^ cells was similarly distributed between CD70^high^ and CD70^low^ populations (**Supplementary Figure 2A**). We then plated the sorted cells on anti-MHCI-coated cover glasses. TIRF imaging confirmed that CD70^high^ cells predominantly formed firework synapses (65.72% ± 11.51%, mean ± SD), while CD70^low^ cells adopted the pancake morphology (82.64% ± 1.02%, mean ± SD; **Figure 2C**).

Helft and colleagues have reported that BMDC cultures may contain a substantial proportion of macrophages (30). To rule out the possibility that one of our populations consisted of macrophages, we evaluated the expression of Zbtb46, a transcription factor selectively expressed in classical dendritic cells (30–32). Comparing Zbtb46 expression in our BMDC cultures to bone marrow-derived macrophage (BMDM) cultures revealed that CD70^low^ and CD70^high^ BMDC populations expressed approximately five times more Zbtb46 than BMDMs (**Figures 2D,E**), with 92.35% (± 1.266% SD) Zbtb46 positive cells (**Supplementary Figure 2B**) and no significant difference of expression between CD70^high^ and CD70^low^ cells (**Supplementary Figure 2C**), confirming their identity as classical dendritic cells. To exclude the possibility that residual activated macrophages contaminated our BMDC preparations, we further characterized the MHCII/CD11c-negative or low populations in CD70^high^ and CD70^low^ cells by assessing Zbtb46 expression. As expected, MHCII^+^ CD11c^+^ cells exhibited higher Zbtb46 expression compared to MHCII/CD11c-negative or low populations, consistent with their dendritic cell identity, with no differences between CD70^high^ and CD70^low^ cells (**Supplementary Figure 2D**). Given the lower Zbtb46 expression in MHCII/CD11c-negative or low cells, we next evaluated their expression of the macrophage-lineage markers CD64 and CD115. Overall, expression of both markers was minimal (**Supplementary Figures 2E, H**). Although a small subset of CD70^high^ MHCII^-^ CD11c^-^ cells displayed relatively higher CD64 or CD115 expression (**Supplementary Figures 2F, I**), these cells represented only a very minor fraction of the total population. Quantitative analysis further confirmed that their frequency did not significantly differ between CD70^high^ and CD70^low^ conditions (**Supplementary Figures 2G, J**). These MHCII/CD11c-low/negative cells most likely represent undifferentiated precursors rather than functional macrophages. Taken together, these results indicate that neither CD70^high^ nor CD70^low^ BMDC populations nor biased by macrophage contamination.

Finally, to test whether the CD70^low^ population merely represents a less mature state, we compared canonical activation markers between the CD70^low^ and CD70^high^ cells following LPS stimulation (1) (**Figure 2F**). Both populations confirmed their mature status by markedly upregulating markers like MHCII, CD80, and CCR7 relative to immature BMDCs. Interestingly, CD70^high^ cells displayed higher levels of CCR7, and CD86 than CD70^low^ cells. This difference in marker expression does not mean one population is an immature precursor to the other. Rather, both subsets are fully mature with subtle differences of maturation marker that delineate functionally specialized subpopulations of activated BMDCs. Given that these populations, once sorted, are committed to forming morphologically distinct and stable pancake or firework synapses for several hours (as shown in **Figure 1**C and 2C), the data strongly supports the conclusion that these are not simply different activation states, but rather two stable and functionally divergent subsets of mature BMDCs.

### Divergent gene expression programs define two functionally distinct dendritic cell subpopulations with unique synapse architectures

**Figure 3:**
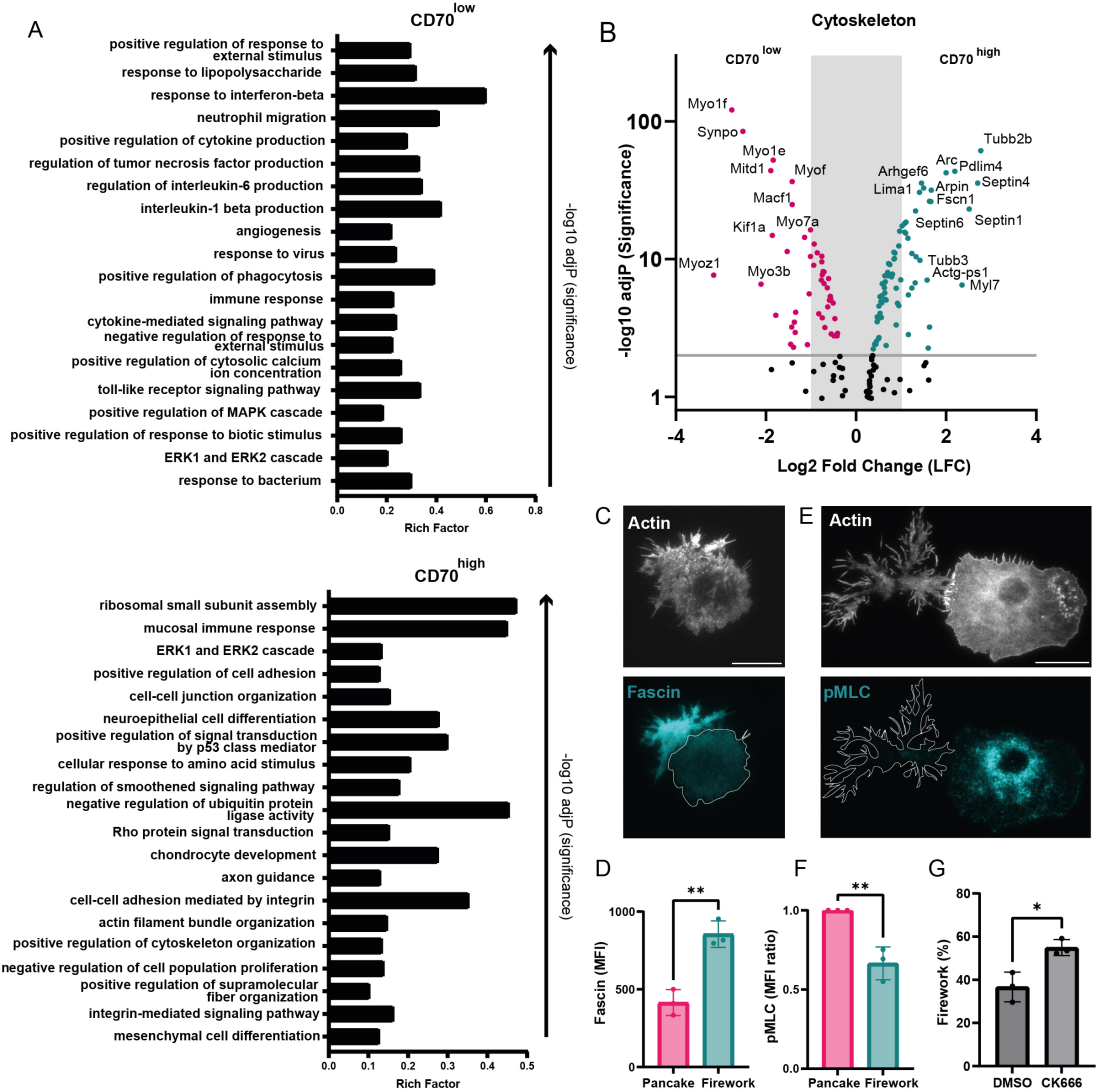
CD70^low^ and CD70^high^ BMDCs display different cytoskeletal signatures. **(A)** Gene ontology (GO) enrichment analysis of RNAseq data from CD70^high^ vs CD70^low^ BMDCs, displayed are the 20 most significantly differentially expressed pathways. **(B)** Volcano plots highlighting differentially expressed genes related to the cytoskeleton (p < 0.01, log2FC > 2). **(C)** TIRF images showing Fascin localization across synapse phenotypes in BMDCs on anti-MHCI-coated glass after 30min adhesion (scale bar: 20 µm). **(D)** MFI quantification of Fascin for each synapse phenotype (n_1_ = 75 cells, n_2_ = 97 cells, n_3_ = 73 cells). **(E)** TIRF images showing phospho-myosin light chain (pMLC) expression in pancake and firework synapses (n=3 biological replicates; scale bar: 20 µm). **(F)** Quantification of MFI of pMLC from synapse phenotypes; MFI normalized to pancake synapses (n_1_ = 143 cells, n_2_ = 323 cells, n_3_ = 62 cells). **(G)** Frequency of firework synapses of BMDCs treated with the Arp2/3 inhibitor CK666 (100 µM) or DMSO control for 1h before synapse formation on anti-MHCI coated glass n_1_ = 266 cells, n_2_ = 239 cells, n_3_ = 290 cells). In **(D, F, G)**, mean +/- SD is shown. Statistical significance was determined using an Unpaired Student’s t-test. *p =< 0.05; **p=< 0.01. For all, n=3 biological replicates.

To define the transcriptional programs that underpin the distinct functional profiles and synapse architectures of CD70^low^ and CD70^high^ BMDCs, we performed bulk RNA-sequencing on the sorted populations. The RNA-seq analysis confirmed that CD70^high^ BMDCs also express higher levels of CD70 mRNA (**Supplementary Figure 3A**), indicating that the differences observed by confocal imaging (**Supplementary Figure 1**) are not merely due to redistribution during synapse assembly, but reflect an inherent difference in total CD70 abundance between pancake and firework cells. Gene Ontology (GO) enrichment analysis confirmed that both CD70^low^ and CD70^high^ cells were fully mature, as upregulated genes are linked to pathogen response (**Figure 3A**). This was consistent with their expression of co-stimulatory markers (**Figure 2F**) and their similar production of the key pro-inflammatory cytokine IL-12, as measured from the supernatant after 48 hours of *in vitro* culture post-sorting (**Supplementary Figure 3B**).

Despite these similarities, the analysis revealed two functionally divergent programs. The CD70^low^ population was enriched for genes involved in specific inflammatory responses, such as IL-6 production. This was confirmed by ELISA measurements of IL-6 secretion in the supernatant of sorted BMDCs after 48 hours in culture, which showed that CD70^low^ cells produced approximately 4.5 times more IL-6 than their CD70^high^ counterparts (33.40 ng/mL ± 6.37 vs. 7.54 ng/mL ± 3.33, mean ± SD; **Supplementary Figure 3C**). In stark contrast, the CD70^high^ population showed a profound enrichment for pathways directly related to cellular structure and motility, including “Rho protein signal transduction”, “actin filament bundle organization”, and “positive regulation of cytoskeleton organization”. This profile was complemented by a survival advantage, as CD70^high^ cultures were also significantly more viable, suggesting they have a longer lifespan (**Supplementary Figure 3D**).

The difference between each subpopulation was confirmed at the level of individual genes (**Figure 3B**). The CD70^high^ firework cells expressed a suite of genes dedicated to building spiky, filamentous protrusions. This includes Fascin-1 (FSCN1), a canonical actin-bundling protein essential for filopodia, and Arhgef6, an activator of the Rho-GTPases that drives these structures. Critically, they also expressed more Arpin, an inhibitor of the Arp2/3 complex (33), which would actively suppress the broad, lamellipodial spreading characteristic of pancakes, since inhibition of branched actin and the Arp2/3 complex has been shown to lead to irregular spreading cells (34). Moreover, the higher expression of Septin 4 and Septin 6 would reinforce this phenotype, as these cytoskeletal components are known to form scaffolds that stabilize the base of actin-based projections (35, 36). Conversely, CD70^low^ cells showed a higher expression of a different cytoskeletal toolkit, including multiple myosins and the microtubule-actin crosslinker Macf1, suggesting a program geared towards managing membrane tension and coordinated spreading. Thus, the transcriptomes reveal two distinct, actively maintained cytoskeletal programs that explain the observed synapse architectures.

To validate the transcriptional findings at the protein and functional level, we performed immunofluorescence staining. In strong agreement with our sequencing data, we confirmed that firework cells expressed significantly higher levels of the filopodial actin-bundler Fascin (**Figures 3C, D**). Conversely, pancake cells exhibited higher myosin IIA activity, measured by phosphorylated myosin light chain (pMLC), which is consistent with a cytoskeletal program geared towards contractility and smooth spreading (**Figures 3E, F**). Finally, to functionally test the implication of the Arp2/3 pathway suggested by the upregulation of its inhibitor Arpin, we pharmacologically blocked the pathway with the small molecule inhibitor CK-666. This treatment was sufficient to significantly increase the proportion of firework synapses, confirming that suppression of Arp2/3-mediated spreading is a key driver of the firework morphology (**Figure 3G**, **Supplementary Figure 3E**). Therefore, from transcription to protein function, our results confirm that the two synapse morphologies are driven by fundamentally different and actively maintained cytoskeletal programs.

### CD70^low^ and CD70^high^ dendritic cells induce a broadly similar early CD8^+^ T cell response

**Figure 4:**
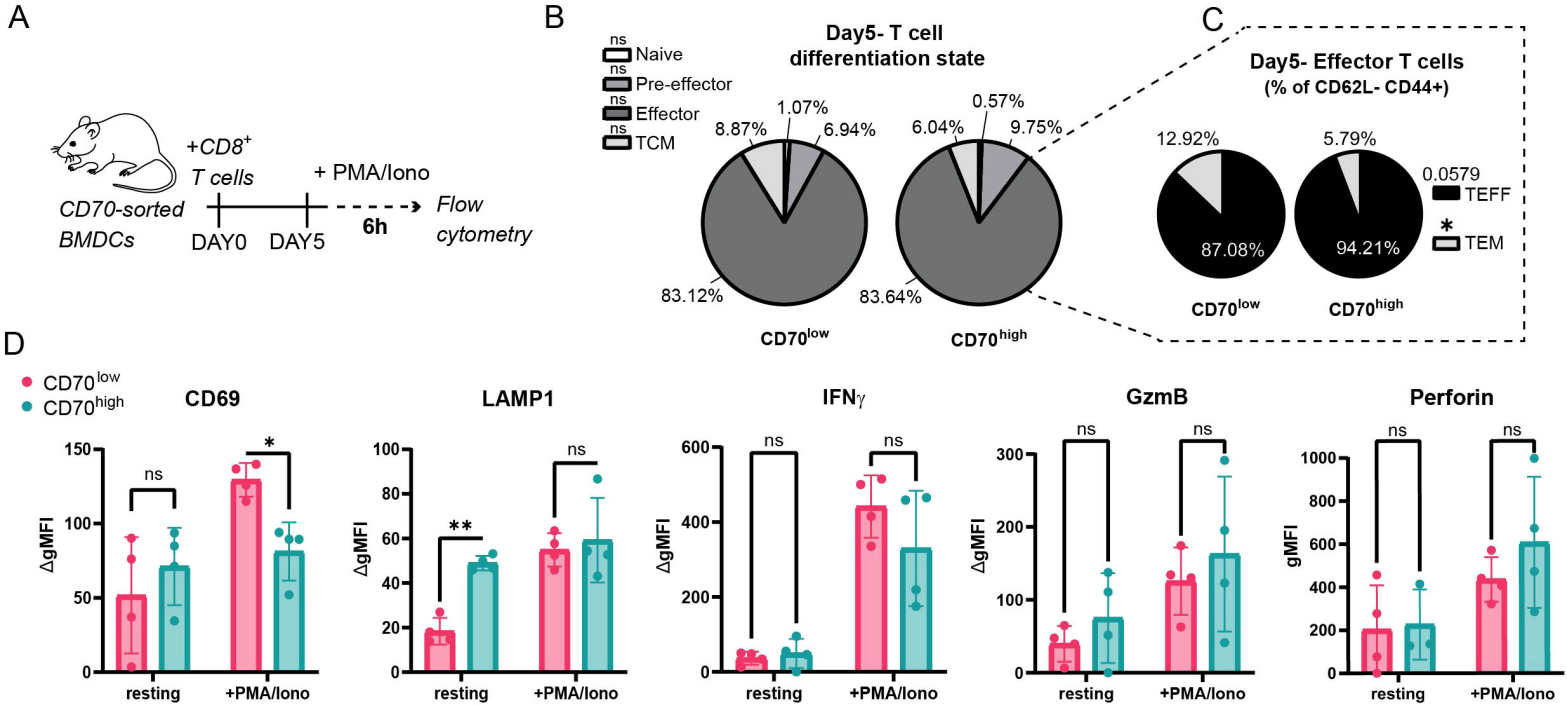
CD70^low^ and CD70^high^ dendritic cells induce a broadly similar early CD8^+^ T cell response. **(A)** Experimental schematic for the primary T cell response assay. Sorted CD70^low^ or CD70^high^ BMDCs were loaded with SIINFEKL peptide and co-cultured with naive OT-I CD8^+^ T cells for 5 days. **(B)** Differentiation state of T cells at day 5, based on CD44 and CD62L expression (n=5 biological replicates). **(C)** Frequencies of effector (TEFF; CD127^-^) and effector memory (TEM; CD127^+^) T cells within the CD44^+^ CD62L^-^ population (n=5 biological replicates). **(D)** Expression of activation and effector molecules in T cells after a 6h restimulation with PMA/ionomycin (n=4 biological replicates). Data are shown as mean ± SD. *p ≤ 0.05; **p ≤ 0.01; ns, not significant. Statistical significance was determined using two-way ANOVA.

To determine if the distinct dendritic cell subpopulations and their synapse architectures differentially prime CD8^+^ T cells, we co-cultured SIINFEKL-loaded CD70^low^ or CD70^high^ BMDCs with naive OT-I transgenic T cells and assessed their activation and differentiation after five days (**Figure 4A**) using flow cytometry (gating strategy in **Supplementary Figure 4**).

At this early time point, the overall T cell response was largely comparable regardless of the stimulating dendritic cell subpopulation. Both dendritic cell populations generated similar proportions of the major T cell memory and effector subpopulations (**Figure 4B**). We did, however, observe a subtle but significant skew within the effector T cell compartment: CD70^low^ dendritic cells favored the generation of effector memory T cells (TEM), while CD70^high^ dendritic cells showed a trend towards more terminal effector cells (TEFF) (**Figure 4C**). To assess effector capacity, we found that upon restimulation, the ability of T cells to produce key cytotoxic molecules (IFNγ, Granzyme B, Perforin) and to degranulate (LAMP1) was equivalent between the groups (**Figure 4D**).

Together, these findings indicate that both dendritic cell populations are potent activators of naïve CD8^+^ T cells, with the distinct synapse types resulting in only minor phenotypic differences during the initial phase of the primary response.

### CD70^high^ and CD70^low^ dendritic cells prime functionally distinct CD8^+^ T cell effector and memory fates

**Figure 5:**
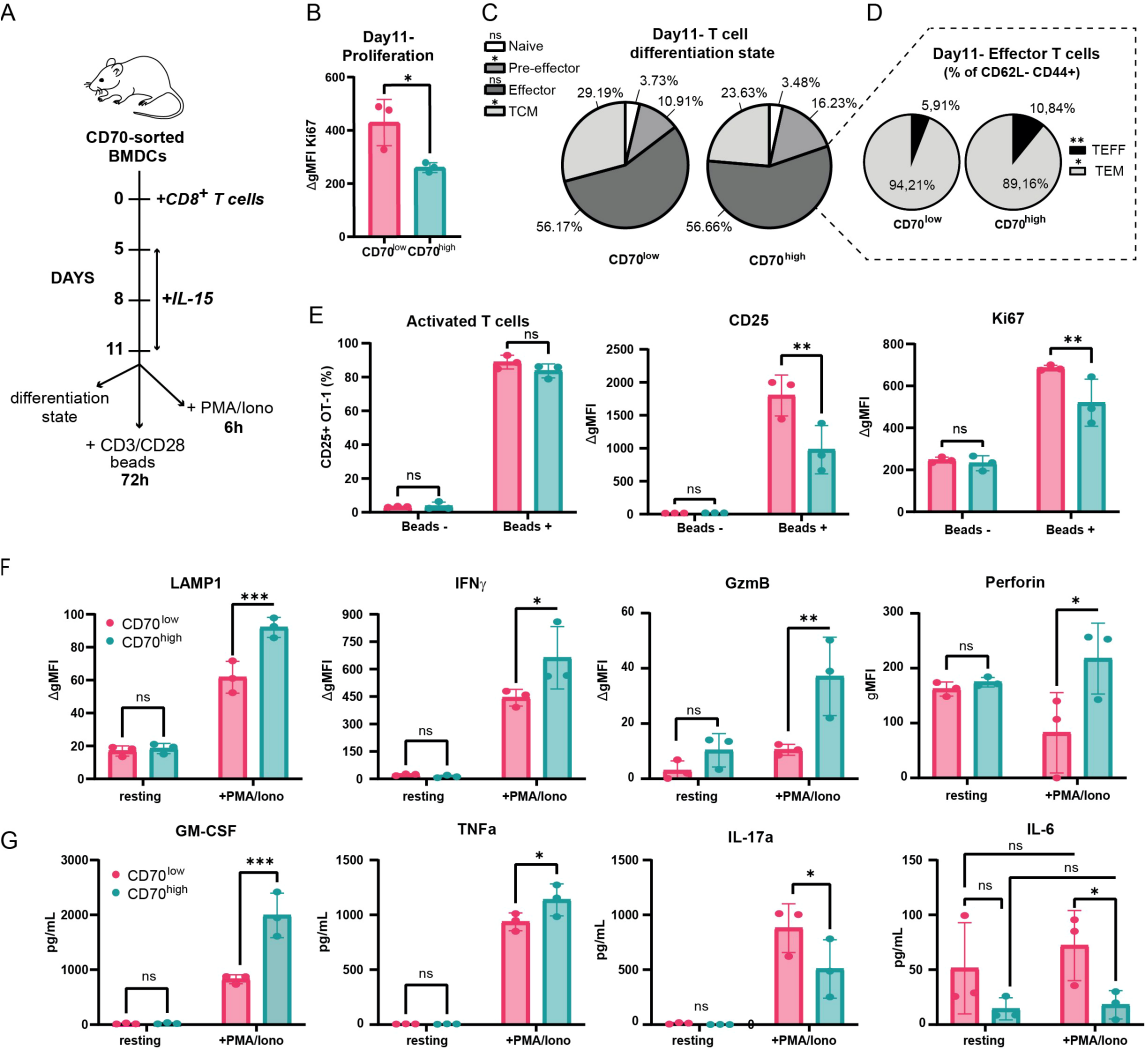
CD70^low^ and CD70^high^ dendritic cells prime distinct long-term CD8^+^ T cell effector and memory fates. **(A)** Experimental schematic for long-term T cell differentiation. Co-cultures were established as in **Figure 4**, with IL-15 added every 3 days from day 5 onwards. On day 11, T cells were tested for their differentiation state and proliferation, restimulated with αCD3/αCD28 beads for 72h or PMA and ionomycin for 6h. **(B)** T cell proliferation at day 11, assessed by Ki67 staining (unpaired Student’s t-test). **(C, D)** T cell differentiation state at day 11, showing distribution of T cell subpopulations. **(C)** and a breakdown of the effector compartment into TEFF and TEM cells **(D)**. **(E)** T cell activation (CD25) and proliferation (Ki67) following bead restimulation. **(F)** Expression of cytotoxic molecules in T cells after 6h of restimulation with PMA/ionomycin on day 11. **(G)** Cytokine secretion profile of restimulated T cells on day 11, measured by multiplex array. Data are shown as mean ± SD (n=3 biological replicates). *p ≤ 0.05; **p ≤ 0.01; ***p ≤ 0.001; ns, not significant. Statistical significance was determined using two-way ANOVA.

Given the initial skew towards different memory precursors, we next sought to determine the long-term functional consequences of CD8^+^ T cell priming by CD70^low^ and CD70^high^ dendritic cells. We assessed T cells on day 11 of co-culture, a time point where memory populations were established with the support of IL-15, added every 3rd day from day 5 (37) (**Figure 5A**). The results revealed a clear divergence in CD8^+^ T cell fate. Notably, CD8^+^ T cells primed by CD70^high^ BMDCs already showed signs of contraction, with significantly lower baseline proliferation (Ki67) compared to their CD70^low^-primed counterparts (**Figure 5B**), consistent with a more terminally differentiated state. This divergence was further reflected in the memory profile, where T cells primed by CD70^low^ BMDCs showed a distinct memory profile, characterized by a higher proportion of central memory (TCM, 29.19% vs. 23.63%) and effector memory (TEM, 94.21% vs. 89.16%) cells (**Figures 5C, D**). Critically, these cells mounted a superior recall response upon restimulation with beads coated with anti-CD3 and anti-CD28, exhibiting significantly higher expression of the activation marker CD25 and the proliferation marker Ki67 (**Figure 5E**).

In contrast, T cells primed by CD70^high^ BMDCs were geared towards a more immediate, terminal effector function. While showing lower recall proliferation, these T cells displayed a superior short-term cytotoxic response upon restimulation with PMA and ionomycin for 6 hours, with significantly higher degranulation (LAMP1) and production of key cytotoxic molecules like Granzyme B, Perforin and IFNγ (**Figure 5F**). To profile the functional polarization of these CD8^+^ T cells more comprehensively, we analyzed a panel of 18 cytokines from the culture supernatants. This confirmed the functional dichotomy: T cells primed by CD70^high^ BMDCs secreted higher levels of the classic pro-inflammatory cytokines TNFα and GM-CSF, indicative of a potent Tc1-like response. Strikingly, T cells primed by CD70^low^ dendritic cells secreted significantly more IL-17A (**Figure 5G**). This suggests they were polarized towards a Tc17-like functional phenotype (38), a lineage associated with long-term memory (39), likely driven by the high levels of IL-6 consistently produced by the CD70^low^ dendritic cells themselves (40) (**Figure 3A**, **Supplementary Figure 3C**).

Taken together, these data support the conclusion that CD70^low^ and CD70^high^ BMDCs promote distinct and durable CD8^+^ T cell differentiation programs. The CD70^low^ dendritic cells generate memory CD8^+^ T cells with a Tc17-like functional profile, while CD70^high^ dendritic cells generate potent but more terminally differentiated Tc1-like effector cells. This suggests a model where the nature of the initial dendritic cell-T cell interaction is critical. It is tempting to speculate that the stable, broad “pancake” synapse of the CD70^low^ BMDC, combined with its unique IL-6 secretion, may create a signaling environment that fosters memory, whereas the dynamic, spiky firework synapse of the CD70^high^ BMDC may delivers a potent, acute signal that drives terminal effector differentiation.

## Discussion

The functional specialization of dendritic cell subsets is a cornerstone of adaptive immunity, ensuring that the nature of the T cell response is tailored to the specific immunological challenge (22). Our study reveals the existence of two stable subpopulations within BMDCs, distinguished by a novel functional criterion: the architecture of the immunological synapses they form. Upon MHCI engagement, dendritic cells assemble either smooth, broadly spreading pancake synapses or synapses with dynamic, spiky firework protrusions. We then demonstrate that this morphological dichotomy correlates with the differential expression of the costimulatory molecule CD70, thereby linking a specific biophysical behavior back to a molecular phenotype. This work therefore expands the concept of dendritic cell heterogeneity, suggesting it encompasses not only the differential expression of surface molecules but also distinct, pre-set cytoskeletal programs that govern the physical nature of the immunological synapse.

It is well-established that the dendritic cell cytoskeleton is highly dynamic and undergoes significant remodeling upon maturation. This process is known to enhance their migratory capacity through lymphoid tissues (41), facilitate the structural organization of the immunological synapse (23, 42, 43), and increase cell stiffness to promote more potent T cell activation (44). Our work suggests that beyond these dynamic adaptations to maturation, distinct dendritic cell subpopulations possess intrinsically different, hardwired cytoskeletal programs that predetermine the very architecture of the synapse they form with T cells.

The cytoskeleton plays a central role on the T cell side of the immunological synapse. It builds the synapse morphology, organizes signaling receptors into functional microclusters, and controls the polarized secretion of cytokines and lytic granules (17, 45–47). The adaptability of this cytoskeletal machinery is evident in the fact that T cells form structurally different synapses depending on the context; they form the classic bull’s-eye architecture with B cells but multifocal synapses with dendritic cells (48) and this diversity extends even to T cell subsets, as Th1 cells form canonical bull’s-eye synapses while Th2 cells assemble multifocal structures (49), although the precise functional purpose of this architectural diversity remains poorly understood.

However, the synapse is a two-sided interface, and emerging evidence indicates that the cytoskeleton of the antigen-presenting cell (APC) is not a passive scaffold but an active participant in programming the T cell response. It has been shown that the biophysical properties of the APC that directly depend on the cytosekeleton, such as cell stiffness, can modulate T cell activation (44, 50), with stiffer dendritic cells generally acting as more potent T cell activators. This principle extends to cytotoxic function, as the mechanical properties of target cells can influence their susceptibility to being killed by cytotoxic T cells (51, 52). Our results build upon this framework by providing direct evidence that distinct, pre-set cytoskeletal programs within dendritic cells are a key determinant of synapse architecture, representing a novel mechanism to shape the instructive signals that program CD8^+^ T cell fate.

Furthermore, our work uncovers a layer of bidirectional communication. The finding that extrinsic CD27 engagement can shift the balance of dendritic cells toward the firework phenotype implies that the T cell is not a passive recipient of information. This supports a model where the CD8^+^ T cell’s own activation state, reflected in its surface CD27 density, can influence the type of synapse the dendritic cell forms, thereby shaping its own subsequent priming. Indeed, the expression of CD27 is rapidly upregulated upon TCR stimulation and is highly expressed on activated and memory T cell populations (11, 53). It is therefore tempting to speculate that a recently activated CD8^+^ T cell, by presenting high levels of CD27, can “demand” a potent, effector-promoting firework synapse from the next dendritic cell it encounters.

The established literature demonstrates that CD70 is indispensable for T cell differentiation, as CD70-deficient DCs fail to generate the expected effector and memory responses (11, 53). Our study does not aim at re-proving this essential role. Instead, we reveal an additional layer of regulation: the existence of two distinct BMDC subpopulations that differ not only in CD70 expression levels but also in their cytoskeletal programs and synapse architectures. The novelty here is not whether CD70 matters, but that differences in its expression correlate with qualitatively different DC states, each coupling a particular synaptic architecture with a defined cytokine profile. This demonstrates that CD70 is not only required in a binary sense (present vs. absent), but that its graded expression can tune the structural and functional landscape of the DC–T cell synapse. In this way, we extend the canonical view of CD70 as a costimulatory switch by showing that it also integrates into broader, architecture-linked differentiation programs of the dendritic cell itself.

Our results further align with the established dual role of the CD27-CD70 axis, where the outcome of CD8^+^ T cell priming is tuned by the magnitude of the costimulatory signal. While CD70 engagement is widely recognized for promoting memory precursor survival (11, 53), it is also a potent driver of clonal expansion and effector differentiation when the signal is strong. Indeed, constitutive CD27/CD70 interaction *in vivo* has been shown to induce the accumulation of effector-type T cells with enhanced IFNγ production (54). In this context, our data suggest that DC heterogeneity acts as a physiological rheostat for this signal. The firework DCs deliver a high-magnitude signal that drives potent Tc1 effector differentiation, consistent with models of strong costimulation. Conversely, the pancake DCs provide the moderated signaling regime necessary to foster the Tc17-like memory lineage. Thus, the synapse architecture may serve to physically enforce these distinct signaling thresholds. We propose that the concentration of CD70 into high-density clusters at the firework synapse increases the local avidity of the interaction, thereby amplifying the effective signal strength delivered to the CD8^+^ T cell to drive effector differentiation. We acknowledge that rigorous quantification of CD70 clustering would be required to support this conclusion and would necessitate higher-resolution approaches, such as PALM or STORM microscopy. In addition, definitive proof that CD70 expression directly and causatively drives this specific cytoskeletal program, rather than acting as a marker for it, would require loss-of-function studies, such as a CD70 knockdown, represent an important future direction.

A key question is whether the synapse architecture or the cytokine milieu is the dominant driver of CD8^+^ T cell fate. Our data suggests this may be a false dichotomy. We propose that the CD70^low^ and CD70^high^ populations represent two distinct and holistic differentiation programs. The pancake program in CD70^low^ cells encompasses not only a cytoskeletal state favoring broad, stable synapses but also a transcriptional signature that includes high IL-6 production. Conversely, the firework program links a filopodia-based synapse with a Tc1-polarizing cytokine profile. Therefore, the architecture and the secreted factors are not independent variables but are co-regulated features of a single cellular identity, working in concert to program a specific CD8^+^ T cell fate. The stable pancake synapse may provide the sustained signaling necessary for memory formation, while the local IL-6 concentration provides the specific polarizing signal for a Tc17-like lineage (38).

Finally, we acknowledge the limitations of our study. Our findings are based on BMDCs, a powerful and widely used *in vitro* system that may not fully recapitulate the vast heterogeneity of primary dendritic cell subpopulations *in vivo*. However, we must emphasize that we use this model not to define novel *in vivo* dendritic cell subpopulations, but rather to uncover a fundamental immunological principle: that a dendritic cell’s costimulatory profile can be intrinsically linked to a specific cytoskeletal program, which in turn dictates synapse architecture and T cell priming. A key future direction will be to employ advanced, dual-color imaging modalities to visualize how these distinct DC cytoskeletal programs are deployed and maintained during a live interaction with a CD8^+^ T cell, an experiment that was beyond the technical scope of the present study. While the precise CD70^low/high^ phenotypes we describe here may be specific to this model, we propose that this mechanistic link between costimulation, cytoskeleton, and synapse shape is a conserved modality of immunological control that is likely employed by various primary dendritic cell subpopulations to fine-tune CD8^+^ T cell responses *in vivo*. In addition, given the cytoskeletal program and predefined CD70 levels before synapse formation, BMDC maturation may be a critical step in pre-configuring cells toward firework or pancake architectures before synapse formation. In this study, we focused on LPS-stimulated BMDCs, however, a broader examination of other TLR pathways, as well as unstimulated BMDCs, would enable a better understanding of how distinct maturation cues influence the balance between synapse types.

In conclusion, our study identifies the interplay between dendritic cell synapse architecture and its associated cytokine profile as a key instructional checkpoint in the programming of CD8^+^ T cell fate. We demonstrate that dendritic cells possess distinct, pre-set cytoskeletal programs that are selected based on the strength of a costimulatory signal, leading to the formation of physically and functionally distinct synapses. This work reframes the immunological synapse not merely as a passive interface for signal exchange, but as a dynamic, programmable structure that actively shapes the outcome of the adaptive immune response.

## Methods

### Cell culture

#### Mice

C57BL/6J and G/OT-1 mice were bred and housed at the University of Konstanz. Male and female mice aged 6 to 14 weeks were used for all experiments. Organ collection was approved by the German Veterinary Authority, and animal experiments were authorized by the Review Board of the Regierungspräsidium Freiburg in compliance with the German Animal Protection Law (T-21/03TFA and T-24/02TFA). Mice were euthanized by gradual CO_2_ asphyxiation in a dedicated chamber.

#### BMDCs

Bone marrow-derived dendritic cells (BMDCs) were generated from wild-type C57BL/6J mice using an established method (55). To obtain bone marrow cells, femurs and tibiae were flushed with phosphate-buffered saline (PBS) using a syringe. The collected cells were then centrifuged at 300 × g for 5 minutes. To remove red blood cells (RBCs), the pellet was treated with 1× RBC lysis buffer (Biolegend, 420301) for 30 seconds at room temperature (RT). The cell suspension was filtered through a 70 μm strainer to eliminate bone fragments and other debris, followed by neutralization with PBS. After another round of centrifugation, the cells were resuspended in R10 culture medium, which included RPMI 1640 (Pan Biotech, PANP04-18500), 2 mM L-glutamine (Pan Biotech, PANP04-82100), 100 U/mL penicillin, 100 μg/mL streptomycin (Pan Biotech, PANP06-07100), 10% heat-inactivated fetal calf serum (iFCS) (Gibco,10270-106), and 50 μM β-mercaptoethanol (Gibco, 31350-010). The cells were then plated at a density of 4 × 10^7^ cells/mL in 10 cm bacteriological Petri dishes containing 10 mL of R10 medium supplemented with 20 ng/mL murine granulocyte-macrophage colony-stimulating factor (GM-CSF; Peprotech, 315-03). On the third day of incubation at 37 °C with 5% CO_2_ in a humidified environment, an additional 10 mL of R10 medium containing 20 ng/mL GM-CSF was added. After 3 more days, half of the culture medium was gently removed from the top and replaced with fresh R10 medium containing 20 ng/mL GM-CSF. By days 8 and 9 of differentiation, cells were classified as immature BMDCs. To promote their maturation, the cells were resuspended in R10 medium supplemented with GM-CSF and 100 ng/mL lipopolysaccharide (LPS) derived from Escherichia coli O111:B4 (Sigma-Aldrich, L4391) and incubated for 20 hours.

#### BMDMs

Bone marrow-derived macrophages were generated from femurs and tibias of C57BL/6 mice. Bone marrow was flushed using a 23-gauge needle and sterile phosphate-buffered saline (PBS), and the resulting cell suspension was centrifuged at 300 × g for 10 minutes at room temperature (RT). Red blood cells were lysed using 1× RBC lysis buffer (BioLegend, Cat# 420301) for 1 minute, followed by filtration through a 70 μm cell strainer. Cells were washed, centrifuged again at 300 × g for 10 min, and counted. On day 0, 1×10^7^ cells were seeded per 96 mm non-treated Petri dish (Greiner) in 10 mL of R10 medium (RPMI 1640 supplemented with 10% heat-inactivated fetal calf serum (FCS), 1% penicillin-streptomycin, 50 μM 2-mercaptoethanol, and 2 mM L-glutamine) containing 10 ng/mL recombinant murine macrophage colony-stimulating factor (M-CSF; PeproTech, Cat# 315-02-10UG). On day 3, an additional 10 mL of R10 medium with 10 ng/mL M-CSF was gently added. On day 6, 10 mL of medium was carefully removed and replaced with fresh R10 medium containing 10 ng/mL M-CSF. BMDMs were considered fully differentiated and ready for use on day 7.

#### Isolation of OT-I T cells

Naïve OT-I CD8+ T cells were isolated from the spleens of G/OT-1 TCR transgenic mice. Spleens were first dissociated in PBS by pressing them through a 70 µm strainer using the plunger of a syringe. The cell suspension was then centrifuged at 300 × g for 10 minutes and resuspended in staining buffer (PBS containing 2% iFCS and 2 mM EDTA (PanReac Aplichem, A3145-0500). The isolation process was completed using the Mouse Naïve CD8a^+^ T Cell Isolation Kit (Miltenyi Biotec, 130-096-543) according to the manufacturer’s instructions. After isolation, the cells were put to R10 culture medium (RPMI 1640 supplemented with 2 mM L-glutamine, 100 U/mL penicillin, 100 μg/mL streptomycin, 10% heat-inactivated fetal calf serum (iFCS), and 50 μM 2-mercaptoethanol) until they were added to the BMDC culture.

### Microscopy

#### Microscopes

Total internal reflection fluorescence (TIRF) microscopy images were acquired using a Leica DMi8 microscope equipped with a 100×/1.47 NA Plan-Apochromat oil immersion objective (Leica Microsystems, Wetzlar, Germany). Fluorescence signals were detected with a DFC9000GTC sCMOS camera (Leica Microsystems).

Confocal microscopy images were acquired using a Nikon Eclipse Ti2-E confocal microscope equipped with a 60X Plan Apo LambdaD immersion oil objective, using 488nm and 640nm excitation lasers and NSPARC system (Nikon).

#### Pseudo-synapse model on glass

Cover Glass 18 mm #1.5 (Marinefeld, 0117580) was functionalized by coating with anti-MHCI antibody (Invitrogen, 16-5999-82) and CD27 recombinant protein (R&D Systems, 574-CD-050) at a concentration of 10 µg/mL in sterile PBS. After incubating for 30 minutes to 1 hour at 37 °C, the cover glasses were washed 3 times with PBS. LPS-matured BMDCs were resuspended at 1 × 10^7^ cells/mL in warm R10 culture medium supplemented with 10 µM HEPES. When indicated, the medium was further supplemented with either 100µM CK666 (*Abcam, ab141231*) or its vehicle, dimethyl sulfoxide (DMSO) (Roth, A994.1) and then incubated for 1 hour at 37 °C.

BMDCs were seeded at 1x10^5^ cells per cover glass in 1 mL of R10 culture medium. The cells were allowed to interact with the functionalized glass for 30 minutes at 37 °C in a 5% CO_2_ atmosphere. After incubation, the cells were fixed with 4% formaldehyde for 20 minutes at 37 °C. The cells were then washed with PBS and permeabilized using 100 µg/mL Lysolecithin (Sigma Aldrich, L5254) for 10 minutes at room temperature (RT), followed by two washes with PBS for 5 minutes each. To prevent non-specific interactions, the samples were blocked with PBS containing 5% bovine serum albumin (BSA, Roth, 3737.1) for 1 hour at RT. The cells were then stained overnight in the dark at 4 °C with anti-CD70 (Abcam, ab223292, 1/50), anti-CD40 (Abcam, ab212058, 1/100), anti-phospho-myosin light chain (pMLC, 3671, Cell Signalling, 1/50), and anti-fascin (Abcam, ab126772, 1/100) in the blocking solution. The samples were washed carefully 3 times with PBS containing 0.5% Tween-20 (Sigma, P1379) for 5 minutes and stained with anti-Rabbit-Alexa-Fluor-488 (Jackson ImmunoResearch, 111-546-046, 1/250), anti-Rabbit-Alexa-Fluor-647 (Jackson ImmunoResearch, 111-606-047) and phalloidin-Alexa Fluor-647 (Invitrogen, A22287, 1/250) for 1 hour in the dark at RT. Finally, the samples were washed 3 times with PBS containing 0.5% Tween-20 for 5 minutes each and imaged using TIRF microscopy the same day.

#### Live imaging

BMDCs were stained in warm media with CellTracker Deep Red dye (Invitrogen, C34565, 1 µM) at 37 °C with 5% CO_2_ for 30 minutes. The cells were then centrifuged at 300 × g for 5 minutes and resuspended in warm medium supplemented with 10 µM HEPES (4-(2hydroxyethyl)-1-piperazine-1-ethanesulfonic acid; Sigma-Aldrich, H0887). Meanwhile, cover glass 18 mm #1.5 was functionalized with anti-MHCI antibody (Invitrogen, 16-5999-82) as described previously. After washing, the cover glasses were placed in the microscope, and 1x10^5^ stained BMDCs were added onto the glass. Image acquisition started immediately. Images were captured every minute for 3 hours at 37 °C in a 5% CO_2_ atmosphere.

#### Image analysis

Images acquired using TIRF microscopy were analyzed with ImageJ (56, v1.54p). Cell outlines were defined using the actin channel, and the mean fluorescence intensity (MFI) of the signal was measured in the channel of interest. When specified, the MFI was normalized to the indicated control (MFI ratio).

To assess whether basal CD70 expression level predicts receptor recruitment to the stimulatory surface, BMDCs were stratified post-acquisition into CD70^high^ and CD70^low^ subpopulations. The median intensity was calculated from confocal z-stacks, pooled across three independent experiments, with CD70^high^ defined as above the median and CD70^low^ as below.

The number of cells was manually counted by three independent counters in a blinded and randomized manner. The counts were averaged and statistically analyzed. Recruitment of CD70 and CD40 to the synapse was quantified from confocal images by measuring mean fluorescence intensity (MFI) at the synapse and normalizing it to whole-cell MFI obtained from a summed Z-projection. The resulting ratio represents the fraction of total marker localized at the synapse.

### Sorted BMDCs

BMDCs were matured with LPS as described previously. The cells were centrifuged at 300 × g for 5 minutes and resuspended at 5 × 10^7^ cells/mL in cold staining buffer (PBS containing 2% iFCS and 2 mM EDTA (Sigma-Aldrich). The cells were then stained in the dark at 4 °C for 30 minutes with anti-CD70-APC antibody (BioLegend, 104610). Afterwards, the cells were washed 3 times with cold staining buffer, and the concentration was adjusted to 2.5 × 10^7^ cells/mL for sorting. The sort was performed using a BD Aria IIu cell sorter. Shortly after sorting, the cells were resuspended in R10 culture medium (RPMI 1640 supplemented with 2 mM L-glutamine, 100 U/mL penicillin, 100 μg/mL streptomycin, 10% heat-inactivated fetal calf serum (iFCS), and 50 μM 2-mercaptoethanol) and incubated at 37 °C with 5% CO_2_. The cells were used for experiments on the same day.

### Cell viability

Once sorted, BMDCs were resuspended at 5x10^5^ cells/mL in R10 culture medium (RPMI 1640 supplemented with 2 mM L-glutamine, 100 U/mL penicillin, 100 μg/mL streptomycin, 10% heat-inactivated fetal calf serum (iFCS), and 50 μM 2-mercaptoethanol) and incubated at 37 °C with 5% CO_2_ for 48 hours. The culture supernatants were then collected and stored at 4 °C for IL-12 and IL-6 quantifications (see section below). The cells were centrifuged at 300 × g for 5 minutes, resuspended in cold staining buffer (PBS containing 2% iFCS and 2 mM EDTA (Sigma-Aldrich)) with 1 µM of Sytox Blue viability dye (Invitrogen, S34857), and respective fluorescence was immediately acquired using flow cytometry.

### Cytokine quantifications of BMDCs 48h cultures

The supernatants from 48-hour cultures were harvested as described previously and used to quantify the release of IL-6 and IL-12 cytokines by BMDCs into the media by ELISA.

The release of IL-12 by BMDCs into the media was assessed by ELISA using the Murine IL-12 Standard ABTS ELISA Development Kit (Peprotech, 900-K97) according to the manufacturer’s instructions, with the exception that detection was performed using Streptavidin-Horseradish Peroxidase (Invitrogen, 434323, 1/2500) and quantified as described previously.

For quantifying the release of IL-6, 96-well plates (Corning Costar, 9018) were coated overnight at 4 °C with an anti-IL-6 capture antibody (R&D Systems, MAB406) at a concentration of 5 µg/mL. The following day, after thorough washing with PBS, the wells were blocked for 1 hour at room temperature (RT) with PBS containing 5% bovine serum albumin (BSA). Samples and IL-6 recombinant protein standard (Peprotech, 200-06) were diluted in PBS, added to the coated wells, and incubated for 1 hour at RT. After incubation, the wells were washed with PBS and incubated with an anti-IL-6 detection antibody (R&D Systems, BAF406) at 2 µg/mL for 1 hour at RT. Detection was performed using Streptavidin-Horseradish Peroxidase (Invitrogen, 434323, 1/2500), incubated for 30 minutes at RT. After multiple washes with PBS containing 0.5% Tween-20, the wells were incubated with TMB substrate for 15 minutes at RT in the dark. The reaction was promptly stopped by adding 1M H_2_SO_4_, and absorbance was read at 450 nm using a Spark Multimode Microplate Reader (Tecan). Quantification was achieved by generating a standard curve from serial dilutions of the recombinant protein and interpolating sample concentrations based on this curve.

### RNA sequencing and data analysis

Total RNA was isolated from each cell-sorted sample stored in 800 µL DNA/RNA Shield (Zymo Research, R1100-50) using a Direct-zol RNA Kit method (Zymo Research, R2053) according to the instruction manual v2.1.0. Notably, a proteinase K treatment was performed on the cells in DNA/RNA Shield according to the instructions in the appendix of the manual prior to the RNA purification steps. The quantity and quality of the purified total RNA were assessed using a Thermo Fisher Scientific Qubit 4.0 fluorometer with the Qubit RNA HS Assay Kit (Thermo Fisher Scientific, Q32855) and an Advanced Analytical Fragment Analyzer System using a Fragment Analyzer RNA Kit (Agilent, DNF-471), respectively. More than 100ng of total RNA was extracted, and all samples had an RNA Quality Number (RQN) or 8.0 or higher. Sequencing libraries were made with 100 ng input RNA using a Revelo mRNA-Seq for MagicPrep NGS kit A & B (Tecan, PN 30186621 & 30186622, respectively) according to the Revelo mRNA-Seq for MagicPrep NGS User Guide (Tecan publication number MO1535, v1). The resulting cDNA libraries were evaluated using a Thermo Fisher Scientific Qubit 4.0 fluorometer with the Qubit dsDNA HS Assay Kit (Thermo Fisher Scientific, Q32854) and an Agilent Fragment Analyzer (Agilent) with a HS NGS Fragment Kit (Agilent, DNF-474), respectively. Pooled cDNA libraries were sequenced 50 bp paired-end using a shared illumina NovaSeq 6000 SP Reagent Kit (100 cycles; illumina, 20028401) on an illumina NovaSeq 6000 instrument. The run produced on average 43.4 million reads/library. The quality of the sequencing run was assessed using illumina Sequencing Analysis Viewer (illumina version 2.4.7), and all base call files were demultiplexed and converted into FASTQ files using illumina bcl2fastq conversion software v2.20. The RNA extractions, quality control assessments, generation of libraries, and sequencing were conducted by the Next Generation Sequencing Platform, University of Bern.

Short reads generated in this study were deposited at the NCBI Sequence Read Archive (SRA, www.ncbi.nlm.nih.gov/sra) and are accessible through the accession number PRJNA1280786. Reads were cleaned from adapters and quality-trimmed with fastp (version 0.23.4, S. 57). Reads were aligned to the mouse transcriptome (ensembl release 112, GRCm39) with salmon (version 1.10.2, (58). One technical replicate (WT1_3_High) was removed because it was consistently different from all other samples and further analyses suggested a potential contamination. For each biological replicate, the three/two technical replicates were summed up. Low and high scatter cells were compared with a general linear model in R with the package DESeq2 (version 1.38.3; (59)). The model accounted for the individual mice (see “paired samples” in the DESeq2 vignette). *P*-values were adjusted for multiple testing (60). Log2 fold-changes were shrunk with the method ashr (61). Genes with an adjusted *P*-value (FDR) below 0.01 and an absolute LFC above 1 were considered differentially expressed.

### GO and KEGG term enrichment

To functionally characterize gene sets, we tested for enrichment of gene ontology (GO) terms and KEGG pathways. GO term enrichment was done with topGO 2.50.0 (62) in conjunction with the GO annotation available through biomaRt (63). The analyses were based on gene counts comparing the gene set of interest with a gene set reference. The analyses were done with the “weight” algorithm with Fisher’s exact test and a minimal node size of 5 (all implemented in topGO). Enrichment of KEGG pathways was tested with clusterProfiler (version 4.6.2, (64) using the gene-to-pathway mappings available through biomaRt (61) and the package org.Mm.eg.db (version 3.16.0). Terms or pathways were identified as significant if the *P*-value was below 0.01.

### T cell coculture with sorted BMDCs

Sorted BMDCs were pulsed for 1 hour at 37 °C with 1 µM SIINFEKL (Sigma, S7951) in warm R10 culture medium (RPMI 1640 supplemented with 2 mM L-glutamine, 100 U/mL penicillin, 100 μg/mL streptomycin, 10% heat-inactivated fetal calf serum (iFCS), and 50 μM 2-mercaptoethanol). After 3 washes with warm media, BMDCs were co-cultured with freshly isolated naïve OT-I CD8^+^ T cells at a 1:10 ratio (BMDCs: T cells) at 37 °C with 5% CO_2_. On days 5 and 8, half of the media was replaced by fresh medium supplemented with 20 ng/mL of IL-15 (Peprotech, 210-15-50UG). At day 5 and 11, cells were subjected to flow cytometric analysis for T subset characterization or resuspended in media for cytotoxicity assessment or restimulation experiments. To evaluate cytotoxic potential, cells were incubated first with CD107a (LAMP-1) antibody or isotype (1:400 and 1:100, respectively) for 1h at 37 °C. Thereafter, 20 ng/mL PMA (Sigma-Aldrich, P1585) and 1 µg/mL ionomycin (Sigma-Aldrich, i3909) as well as Brefeldin A (Biolegend, 420601, 1/1000) were added for another 5 hours at 37 °C with 5% CO_2_. Antibodies staining surface and intracellular markers were analyzed by flow cytometry (see Flow cytometry section). Restimulation was performed on day 11 by adding Dynabeads™ Mouse T-Activator CD3/CD28 stimulation beads to T cells at a ratio of 1:1 (Thermo,11452D). Cells were further incubated for 3 days at 37 °C with 5% CO_2_ before being analyzed by flow cytometry. To assess cytokine release, cells were incubated 20 ng/mL PMA (Sigma-Aldrich, P1585) and 1 µg/mL ionomycin (Sigma-Aldrich, i3909) for 6 hours at 37 °C with 5% CO_2_ and culture supernatant was collected and conserved at -20 °C before being sent for analysis to Eve Technologies (Calgary, AB), and analyzed using the multiplex cytokine array Mouse High Sensitivity T-Cell Discovery Array 18- plex (MDHSTC18).

### Flow cytometry

Surface staining for BMDCs was carried out using the following antibodies: anti-MHCII-FITC (Biolegend, 107605, 1/500), anti-CD11c-BV421 (Biolegend, 117330, 1/200), CD11c-PE/Dazzle594 (Biolegend, 117347, 1/150), anti-CD70-APC (Biolegend, 104610, 1/100), anti-CD40-PE (Invitrogen, 12040182, 1/200), anti-CD80-PE (BD, 553769, 1/100), anti-CD86-PE (BD, 553769, 1/100), anti-CCR7-PE (eBioscience, 12-1971-83, 1/25), anti-CD64-BV605 (Biolegend, 139323, 1/100) and anti-CD115-BV421 (Biolegend, 135513, 1/100). Briefly, BMDCs were centrifuged at 300 × g for 5 minutes and resuspended in cold staining buffer (PBS containing 2% iFCS and 2 mM EDTA, Sigma-Aldrich). Cells were incubated with the antibodies at 4 °C in the dark for 30 minutes, then washed thoroughly in cold staining buffer before flow cytometry analysis. Intracellular staining for Zbtb46 was performed using the FOXP3 Intracellular Kit (Biolegend, 411403) following the manufacturer’s protocol, with anti-Zbtb46-PE (BD Biosciences, 565832) and IgG1κ-PE (BD Biosciences, 554685) antibodies. Fluorescent signals were acquired using an LSR II flow cytometer, and data were analyzed with FlowJo V10 software (Tree Star, Ashland, OR, USA).

Cultured OT-I CD8^+^ T cells were stained with 10 µg/ml FC receptor block antibodies (Biolegend, 101330) for 10 minutes at 4 °C. Upon washing 1x with PBS, T cells were incubated with NIR Zombie fixable viability dye solution (Biolegend, 423105, 1/1000) for 15 minutes at 4 °C. Thereafter, surface staining of OT-I T cells was carried out using the following antibodies for surface staining: anti-TCR-Vα2-SB780 (Invitrogen, 78-5812-82, 1/100), anti-TCR-Vα2-PerCp-Cy5.5 (Biolegend, 127813, 1/100), anti-CD44-AF488 (Biolegend, 103016, 1/100), anti-CD62L-BV650 (Biolegend, 104453, 1/100), anti-CD127-PE (Biolegend, 135010, 1/100), anti-CD69-BV650 (Biolegend, 104541, 1/100), anti-LAMP-1-BV421 (Biolegend, 121617, 1/50), anti-CD25-FITC (Biolegend, 101908, 1/100). Intracellular & intranuclear antibodies comprised: anti-IFN-γ-PE (Biolegend, 505808, 1/100), anti-GzmB-FITC (Biolegend, 515403, 1/50), anti-Perforin (Biolegend, 154303, 1/100), anti-Ki67-BV421 (Biolegend, 151208, 1/100). Respective isotype controls were used for both surface & intracellular staining.

Briefly, T cells were centrifuged at 300 × g for 5 minutes and resuspended in cold staining buffer (PBS containing 2% iFCS and 2 mM EDTA, Sigma-Aldrich). Cells were incubated with the surface antibodies at 4 °C in the dark for 15 minutes, then washed thoroughly in cold staining buffer before flow cytometry analysis or intracellular staining. Intracellular cytokine staining was performed using the BD Cytofix/Cytoperm™ Fixation/Permeabilization Kit (BD, 554714) and intranuclear transcription factor staining via FOXP3 Fix/Perm Buffer Set (Biolegend, 421403) following manufacturer’s instructions. Ultimately, 5,000 events of viable T cells were measured on an LSRFortessa device and data analyzed using the Kaluza Analysis Software (Beckman Coulter).

### Statistical analysis

Statistical analyses and data visualization were performed using Prism v10 software (GraphPad, San Diego, CA, USA). Differences between groups were evaluated with unpaired or paired Student’s t-tests, or for comparisons involving more than two groups, one-way or two-way analysis of variance (ANOVA) with uncorrected Fischer’s LSD. A significance level of 5% was set. The criteria for significance were as follows: not significant (n.s.), for p > 0.05, * for p ≤ 0.05, ** for p ≤ 0.01, *** for p ≤ 0.001, and **** for p ≤ 0.0001.

## Data Availability

Raw data supporting the findings of this study are deposited in Zenodo (http://10.5281/zenodo.17101226). Due to large file sizes, some microscopy image datasets are available from the authors upon reasonable request and will be shared without restriction.

## Supplementary material

Supplementary Figure 1 CD70 is sequestered in intracellular compartments and recruited to the synapse upon CD27 engagement. **(A)** Representative 3D confocal images of BMDCs on glass coated with anti-MHCI (aMHCI) alone (left) or together with CD27 (right). The main panels show the XY view, with corresponding orthogonal views (XZ and YZ) displaying CD70 localization. Note the largely intracellular staining in the absence of CD27 and the recruitment to the plasma membrane at the cell-glass interface in the presence of CD27 (n=3 biological replicates, scale bar: 20 µm). **(B)** Representative confoca side-views showing CD40 localization in BMDCs. **(C)** Quantification of CD40 recruitment to the synapse measured measure by confocal microscopy. Briefly, CD40 mean fluorescence intensity (MFI) was quantified at the synapse and normalized by the CD40 MFI in the whole cell (n_1_ = 45 cells, n_2_ = 42 cells, n_3_ = 38 cells). Data are shown as mean ± SD (n=3 biological replicates). ns, non-significant. Statistical significance was determined using two-way ANOVA.

Supplementary Figure 2 BMDCs are bona fide DCs. **(A)** Repartition of BMDCs sub-population based on their expression of MHCII, CD11c and CD70 expression. Statistical significance was determined using two-way ANOVA. **(B)** Quantification of percentage of cells expressing Zbtb46 in our BMDC cultures. **(C)** Quantification of median fluorescence intensity (MFI) of Zbtb46 in CD70^low^ and CD70^high^ BMDCs. Statistical significance was determined using an Unpaired Student’s t-test. **(D)** Quantification of median fluorescence intensity (MFI) of Zbtb46 in BMDCs sub-population based on their expression of MHCII, CD11c and CD70 expression. Statistical significance was determined using two-way ANOVA. **(E-G)** Expression of CD64 in BMDCs cultures. Representative histogram **(E)** and median fluorescence intensity (MFI) quantification **(F)**, two-way ANOVA) in BMDCs depending on their MHCII, CD11c and CD70 expression, and **(G)**, Unpaired Student’s t-test) the percentage of CD70^low^ and CD70^high^ cells expressing CD64 while being MHCII- and CD11c-. (H-J) Expression of CD115 in BMDCs cultures. Representative histogram **(H)** and median fluorescence intensity (MFI) quantification **(I)**, two-way ANOVA) in BMDCs depending on their MHCII, CD11c and CD70 expression, and **(G)**, Unpaired Student’s t-test) the percentage of CD70^low^ and CD70^high^ cells expressing CD115 while being MHCII- and CD11c-. Data are shown as mean ± SD (n=3 biological replicates). **p ≤ 0.01; ****p≤ 0.0001; ns, non-significant.

Supplementary Figure 3 CD70^low^ and CD70^high^ BMDCs exhibit distinct cytokine secretion profiles and viability. **(A)** Volcano plots highlighting differentially expressed genes after sorting cells on CD70 high or low expression before performing RNA-sequencing(p < 0.01, log2FC > 2), (n=3 biological replicates). **(B-D)** Sorted CD70^low^ and CD70^high^ BMDCs were cultured for 48h. Supernatants were analyzed by ELISA for IL-12 (A) and IL-6 **(B)** secretion, and cell viability was assessed by flow cytometry **(C, E)** TIRF images of F-actin in BMDCs forming synapses on anti-MHCI-coated glass. Cells were pre-treated for 1h with the Arp2/3 inhibitor CK666 (100µM) or DMSO as a control. Two representative images per condition are displayed. Note the prevalence of the firework phenotype upon CK666 treatment. Data are shown as mean ± SD (n=3 biological replicates). Statistical significance was determined using an Unpaired Student’s t-test. *p ≤ 0.05; **p ≤ 0.01; ns, not significant. Scale bar: 100 µm.

Supplementary Figure 4 Phenotypic analysis of T cells during primary activation. Representative flow cytometry gating strategy to distinguish effector (TEFF; CD44^+^ CD62L^-^ CD127^-^) and effector memory (TEM; CD44^+^ CD62L^-^ CD127^+^) T cells based on CD44, CD62L, and CD127 expression. T cells were pre-gated for viable TCR-Vα2 expressing T cells.

Supplementary Movie 1 Pancake and firework synapses are stable over time. Representative time-lapse TIRF imaging of a live bone marrow–derived dendritic cell (BMDC) illustrating the formation and stability of two distinct immunological synapse patterns: pancake and firework synapses. Time is indicated in hours and minutes (hh:mm). Cells were stained using CellTracker Deep Red dye (n=3 biological replicates, scale bar = 20μm).
