# Supplementary figures and images for: Distinct dendritic cell cytoskeletal programs dictate synapse architecture and CD8+ T cell fate
Source: Front Immunol. 2026 Apr 1;17:1716644. doi: 10.3389/fimmu.2026.1716644 (PMC13078997; doi:10.3389/fimmu.2026.1716644)

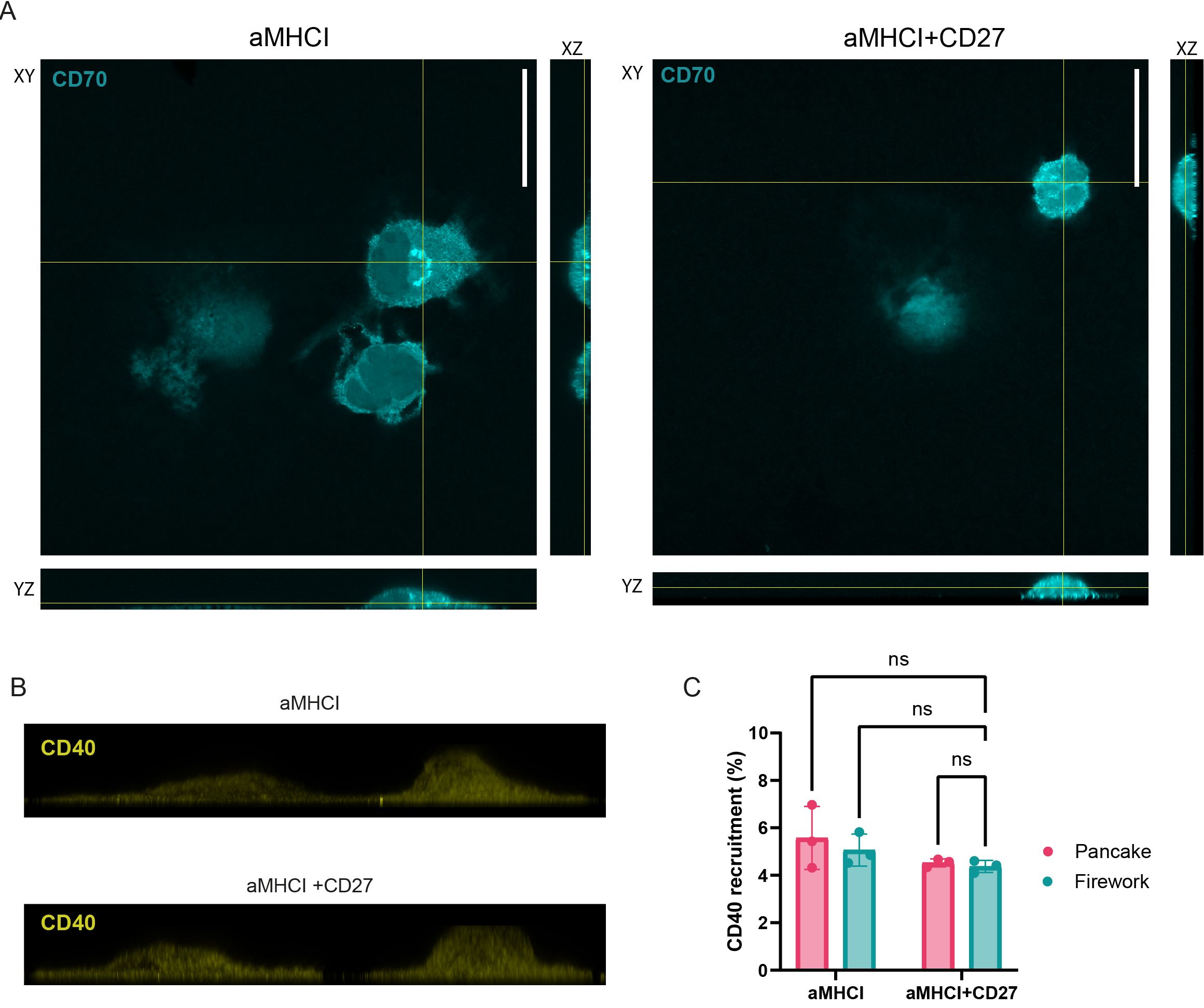

Supplement: Supplementary Figure 1 — CD70 is sequestered in intracellular compartments and recruited to the synapse upon CD27 engagement. (A) Representative 3D confocal images of BMDCs on glass coated with anti-MHCI (aMHCI) alone (left) or together with CD27 (right). The main panels show the XY view, with corresponding orthogonal views (XZ and YZ) displaying CD70 localization. Note the largely intracellular staining in the absence of CD27 and the recruitment to the plasma membrane at the cell-glass interface in the presence of CD27 (n=3 biological replicates, scale bar: 20 µm). (B) Representative confoca side-views showing CD40 localization in BMDCs. (C) Quantification of CD40 recruitment to the synapse measured measure by confocal microscopy. Briefly, CD40 mean fluorescence intensity (MFI) was quantified at the synapse and normalized by the CD40 MFI in the whole cell (n1 = 45 cells, n2 = 42 cells, n3 = 38 cells). Data are shown as mean ± SD (n=3 biological replicates). ns, non-significant. Statistical significance was determined using two-way ANOVA. [file Image1.jpeg]

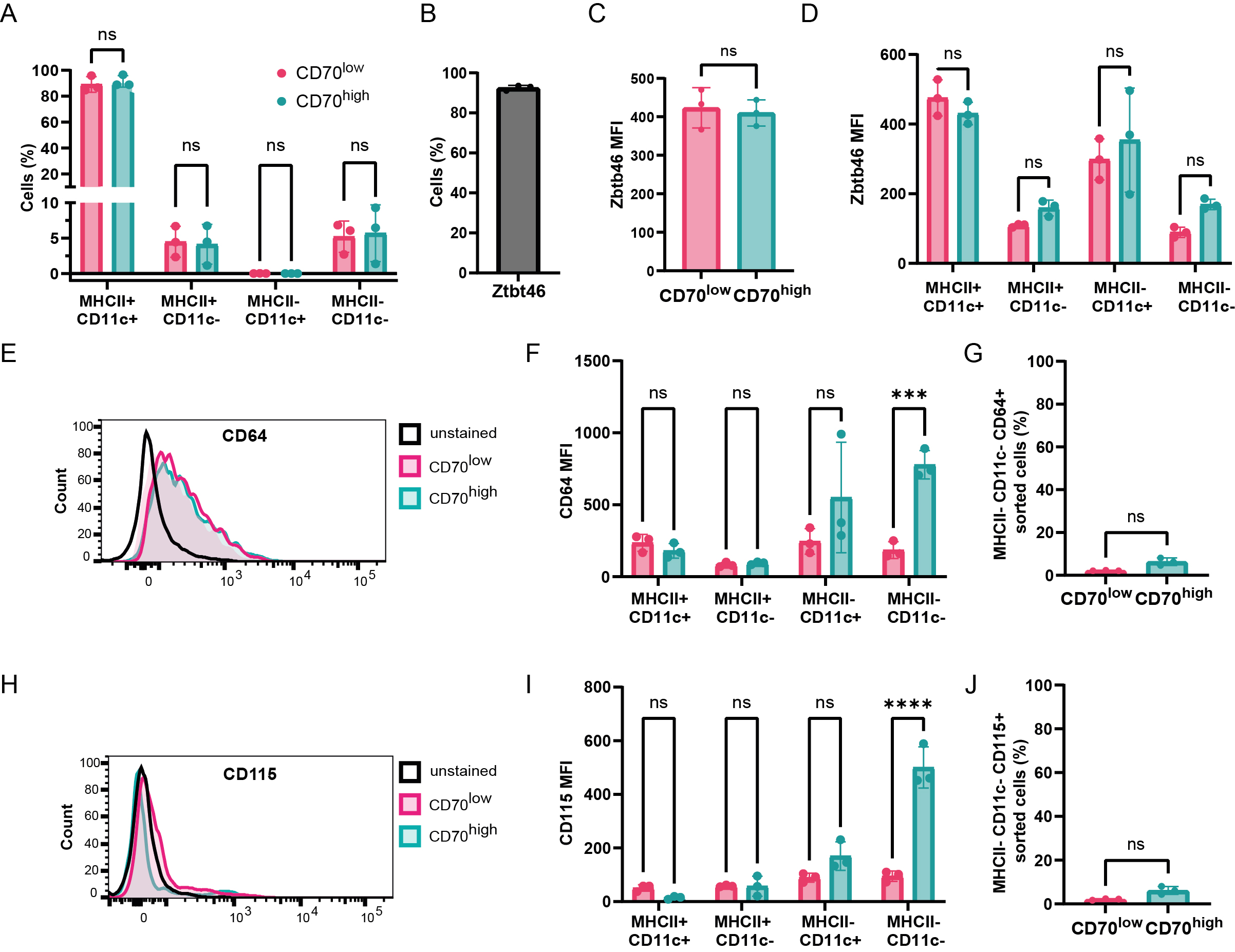

Supplement: Supplementary Figure 2 — BMDCs are bona fide DCs. (A) Repartition of BMDCs sub-population based on their expression of MHCII, CD11c and CD70 expression. Statistical significance was determined using two-way ANOVA. (B) Quantification of percentage of cells expressing Zbtb46 in our BMDC cultures. (C) Quantification of median fluorescence intensity (MFI) of Zbtb46 in CD70low and CD70high BMDCs. Statistical significance was determined using an Unpaired Student’s t-test. (D) Quantification of median fluorescence intensity (MFI) of Zbtb46 in BMDCs sub-population based on their expression of MHCII, CD11c and CD70 expression. Statistical significance was determined using two-way ANOVA. (E-G) Expression of CD64 in BMDCs cultures. Representative histogram (E) and median fluorescence intensity (MFI) quantification (F), two-way ANOVA) in BMDCs depending on their MHCII, CD11c and CD70 expression, and (G), Unpaired Student’s t-test) the percentage of CD70low and CD70high cells expressing CD64 while being MHCII- and CD11c-. (H-J) Expression of CD115 in BMDCs cultures. Representative histogram (H) and median fluorescence intensity (MFI) quantification (I), two-way ANOVA) in BMDCs depending on their MHCII, CD11c and CD70 expression, and (G), Unpaired Student’s t-test) the percentage of CD70low and CD70high cells expressing CD115 while being MHCII- and CD11c-. Data are shown as mean ± SD (n=3 biological replicates). **p ≤ 0.01; ****p≤ 0.0001; ns, non-significant. [file Image2.jpeg]

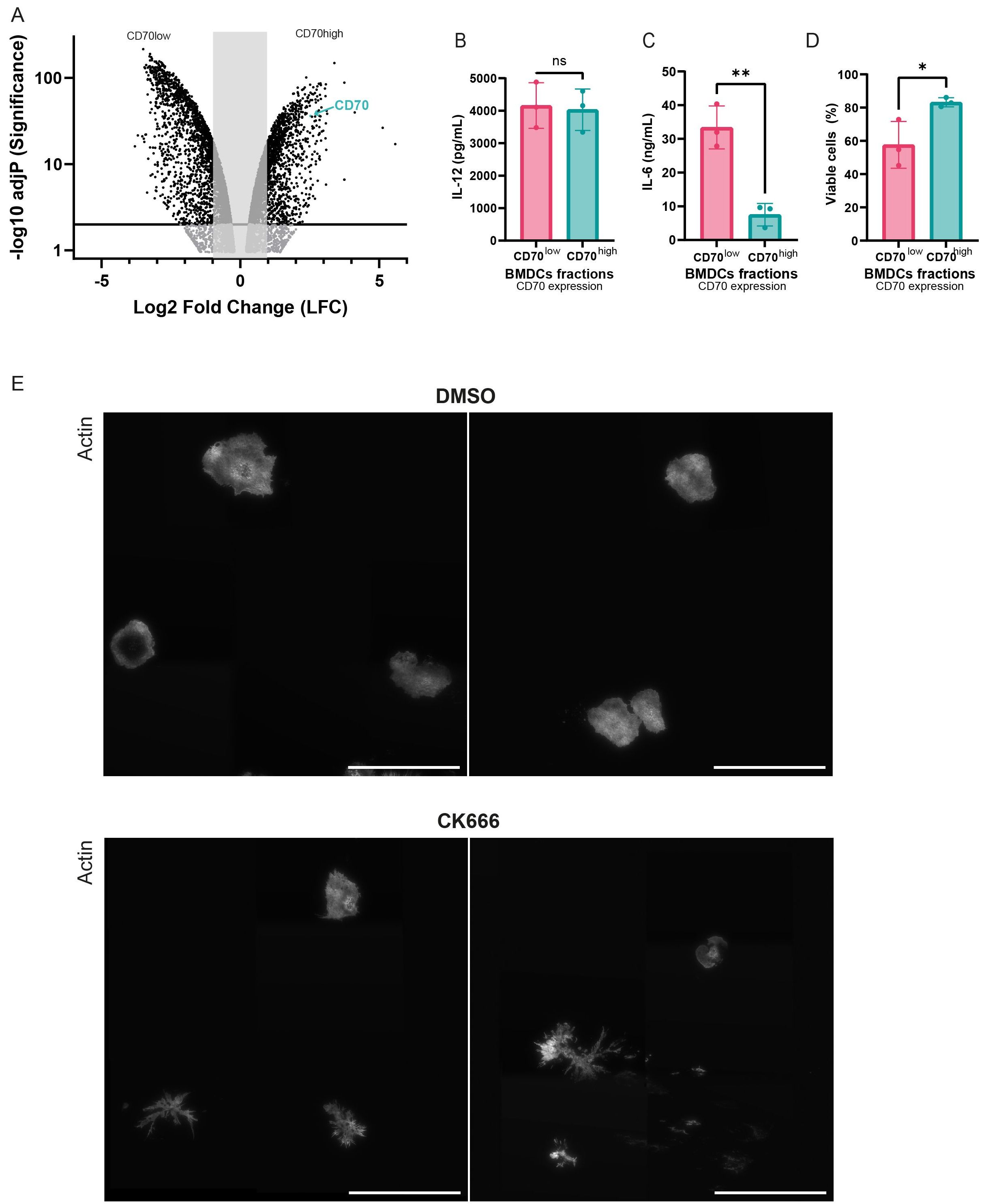

Supplement: Supplementary Figure 3 — CD70low and CD70high BMDCs exhibit distinct cytokine secretion profiles and viability. (A) Volcano plots highlighting differentially expressed genes after sorting cells on CD70 high or low expression before performing RNA-sequencing(p < 0.01, log2FC > 2), (n=3 biological replicates). (B-D) Sorted CD70low and CD70high BMDCs were cultured for 48h. Supernatants were analyzed by ELISA for IL-12 (A) and IL-6 (B) secretion, and cell viability was assessed by flow cytometry (C, E) TIRF images of F-actin in BMDCs forming synapses on anti-MHCI-coated glass. Cells were pre-treated for 1h with the Arp2/3 inhibitor CK666 (100µM) or DMSO as a control. Two representative images per condition are displayed. Note the prevalence of the firework phenotype upon CK666 treatment. Data are shown as mean ± SD (n=3 biological replicates). Statistical significance was determined using an Unpaired Student’s t-test. *p ≤ 0.05; **p ≤ 0.01; ns, not significant. Scale bar: 100 µm. [file Image3.jpeg]

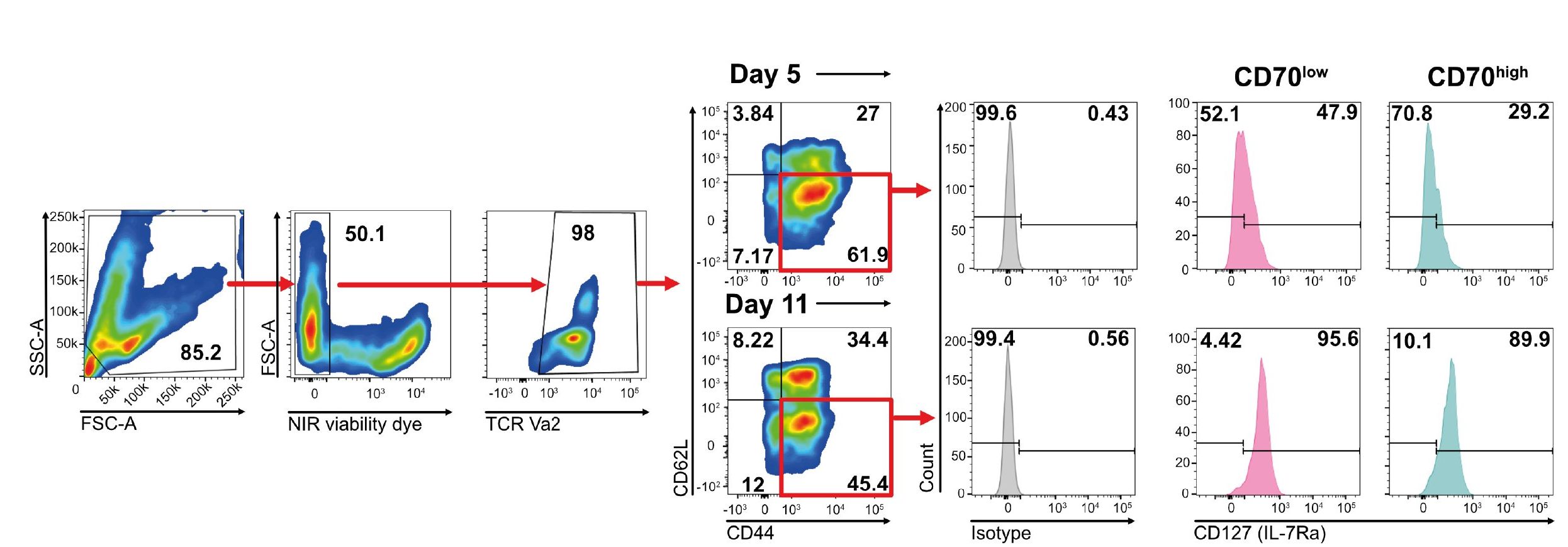

Supplement: Supplementary Figure 4 — Phenotypic analysis of T cells during primary activation. Representative flow cytometry gating strategy to distinguish effector (TEFF; CD44+ CD62L- CD127-) and effector memory (TEM; CD44+ CD62L- CD127+) T cells based on CD44, CD62L, and CD127 expression. T cells were pre-gated for viable TCR-Vα2 expressing T cells. [file Image4.jpeg]
